# Using Machine Learning for Pharmacovigilance: A Systematic Review

**DOI:** 10.3390/pharmaceutics14020266

**Published:** 2022-01-23

**Authors:** Patrick Pilipiec, Marcus Liwicki, András Bota

**Affiliations:** 1 Embedded Intelligent Systems Lab, Department of Computer Science Electrical and Space Engineering, Luleå University of Technology, 97187 Luleå, Sweden; patrick.pilipiec@maastrichtuniversity.nl (P.P.); marcus.liwicki@ltu.se (M.L.); 2 School of Business and Economics, Maastricht University, Tongersestraat 53, 6211 LM Maastricht, The Netherlands

**Keywords:** pharmacovigilance, adverse drug reactions, ADRs, computational linguistics, machine learning, public health, user-generated content

## Abstract

Pharmacovigilance is a science that involves the ongoing monitoring of adverse drug reactions to existing medicines. Traditional approaches in this field can be expensive and time-consuming. The application of natural language processing (NLP) to analyze user-generated content is hypothesized as an effective supplemental source of evidence. In this systematic review, a broad and multi-disciplinary literature search was conducted involving four databases. A total of 5318 publications were initially found. Studies were considered relevant if they reported on the application of NLP to understand user-generated text for pharmacovigilance. A total of 16 relevant publications were included in this systematic review. All studies were evaluated to have medium reliability and validity. For all types of drugs, 14 publications reported positive findings with respect to the identification of adverse drug reactions, providing consistent evidence that natural language processing can be used effectively and accurately on user-generated textual content that was published to the Internet to identify adverse drug reactions for the purpose of pharmacovigilance. The evidence presented in this review suggest that the analysis of textual data has the potential to complement the traditional system of pharmacovigilance.

## 1. Introduction

In drug development, there exists a strong tension between accessibility and safety. While drugs can effectively cure diseases and improve life [1], the required process of research and development of drugs is expensive, and pharmaceutical companies have a high stake in yielding a profit on their investment [2]. This increases the urgency to make effective drugs available to the public. In contrast, medicines can also induce adverse drug reactions (ADRs) that may result in mortality, and the identification of such reactions demands thorough and time-consuming testing of the drug’s safety, drastically increasing the time-to-market of new drugs [3]. In fact, the potential consequences of ADRs are significant. In the European Union (EU), five percent of hospital admissions and almost 200,000 deaths were caused by ADRs in 2008, and the associated societal cost totaled EUR 79 billion [4].

A system that applies tools and practices from the research field of pharmacovigilance was introduced to alleviate this tension [5]. This system performs ongoing monitoring of ADRs of existing drugs [5]. It also minimizes the time-to-market of effective drugs, and it allows their long-term safety post market authorization to be continuously examined [6]. Overall, pharmacovigilance is the cornerstone in the regulation of drugs [1]. The traditional system that applies pharmacovigilance is very expensive and often fails to monitor ADRs experienced by users if these are not reported to the authorities, pharmaceutical companies, or medical professionals [6,7]. The reporting of these ADRs is important because it may help to protect public health [1].

In today’s society, many people share personal content on social media [8,9,10]. An abundance of studies have already demonstrated that user-generated content can be used accurately for remote sensing, among others, to gauge public health [11,12,13,14,15,16,17,18,19,20,21,22,23,24,25,26,27,28,29,30]. This naturally raises the valid question whether user-generated textual content can also be analyzed for the purpose of pharmacovigilance. Such automated analysis may provide a cheap and efficient supplement to the expensive and time-consuming traditional methods for pharmacovigilance, and it may also include first-hand experiences about ADRs from users that were not reported to the authorities, pharmaceuticals, or medical professionals. Although various studies [31,32,33,34,35,36,37,38,39,40,41] were conducted that investigated the suitability of natural language processing (NLP) for pharmacovigilance, to our awareness, no systematic review has yet been conducted that aggregated the reported evidence or assessed the quality of those studies.

To address this research gap, the purpose of this study is to review the existing evidence on, and the effectiveness of natural language processing to understand user-generated content for the purpose of pharmacovigilance. According to our review, it is worthwhile to analyze user-generated content that has already been published to the Internet, to proactively and automatically identify ADRs, without relying on users to actively report those cases to the authorities, pharmaceutical companies, or medical professionals.

## 2. Background

A severe limitation in the process of bringing new drugs to market is the potential of drugs to cause ADRs. While pre-clinical and clinical studies include testing drug safety and potential ADRs, only a total of a few hundreds or thousands of participants are included in these studies [1]. In addition, these studies are performed under controlled clinical conditions that may not represent every real-world situation or circumstance [1]. Therefore, not all ADRs may have been identified prior to making the drug generally available [3]. As long as the benefits outweigh potential costs, it is generally considered unethical to withhold the general public from using an effective drug at this stage, thus accepting that some people may develop ADRs in the future.

### 2.1. Traditional Approaches

To counteract the limitations of pre-clinical and clinical testing, existing drugs on the market are constantly being monitored for safety and ADRs [3]. The long-term monitoring of existing drugs is crucial, because potential ADRs, interactions, and other risk factors, may only emerge many years or even decades after the drug initially received market authorization [3].

The long-term monitoring of drug safety beyond market authorization is named pharmacovigilance [5], which is defined by the WHO as “the science and activities relating to the detection, assessment, understanding and prevention of adverse effects or any other medicine-related problem” [3]. As such, the application of tools and practices from pharmacovigilance by public health authorities results in a pro-active system that is intended to promote and protect public health [1]. It involves a wide array of activities, including data collection about drug safety, obligating pharmaceuticals and medical professionals to report ADRs, inviting patients to report experiences with drugs, and the detection of signals that may indicate drug safety issues [6]. There are, however, significant costs associated with the processing and administration of the reported cases of ADRs [7]. In addition, the current system of collecting data to monitor drug safety is suboptimal because end-users are not obliged to report cases of ADRs [6].

### 2.2. Improving Pharmacovigilance Using Natural Language Processing

In the preceding 15 years, many technological innovations have enabled the storage, processing, and analysis of big data [42,43,44]. In particular, with the emergence of Web 2.0 and social media platforms, there has been a significant increase of user-generated content that is published to the Internet [8,9,10]. Among others, vast amounts of textual data are generated on blogs, forums, and social media [45]. Similarly, there have been significant developments in artificial intelligence that resulted in powerful methods and algorithms for NLP [46], which enabled the processing and understanding of human-generated text [45,47]. This opened new opportunities for mining social media and analyzing texts [48]. In recent years, these fields experienced significant innovations [49].

Text mining is frequently defined as the analysis of textual data, such as unstructured or semi-structured text, with the purpose to extract hidden patterns and information [45]. As such, it combines data mining with NLP [43]. Text mining has emerged from a need to analyze large amounts of text containing human language, which can be mined for insights that facilitate data-driven decision-making [45]. However, many standard data mining techniques cannot be applied to unstructured textual data. Therefore, text mining is applied as pre-processing for unstructured data [50,51], e.g.,:

- tokenization: the separation of text into smaller units, like words, characters, or sub-words (n-grams);
- transformation of cases, such as uniform lowercasing or uppercasing;
- stop word removal: the removal of words carrying very little meaning; and such as pronouns;
- reducing inflected words to their word stem (stemming).

Once text mining has been applied to extract structured data from a semi-structured or unstructured source, conventional data mining algorithms can subsequently be used to process and analyze these structured data further to yield the valued insights [47]. The complexity that is involved with analyzing unstructured textual data and in particular its irregularities, makes the process of text mining a difficult area in artificial intelligence [52].

The applications of text mining are numerous, and include:

- assigning affective states to text (sentiment analysis) [43];
- the discovery of associations between words or other tokens [53];
- the summarization of documents [43];
- clustering texts according to some similarity measurement [54,55];
- classification of text into various categories [56,57];
- predicting words or other lexical units (as part of a word processor or chatbot) [58,59]; and
- the extraction of concepts, entities, and the relationships between them [43].

Among others, NLP was used to monitor public health, such as surveilling allergies [20,24,26], depressions [21,22,30], suicide-related thoughts and conversations [11,16,27], obesity [13,17], marijuana and drug abuse [12,25,28,29], tobacco and e-cigarettes [14,18,19,23], and to gauge public health concerns [15].

## 3. Materials and Methods

This systematic review was guided by the Preferred Reporting Items for Systematic Reviews and Meta-Analyses (PRISMA) guidelines [60,61]. However, most of the reviewed papers do not contain controlled trials, comparable statistical analysis, or methodology, making it impossible to apply the complete PRISMA 2020 checklist to this review. Therefore, we only applied items on the checklist if they were applicable, and thus our review does not conform completely to the guideline. The quality of this systematic review was evaluated using the PRISMA Checklist in Appendix A.

### 3.1. Search Strategy

To cover all related disciplines, a broad selection of databases was made that included PubMed, Web of Science, IEEE Xplore, and ACM Digital Library. These databases were selected because they index studies in a wide range of fields. Specifically, PubMed was included because it predominantly indexes research in the field of public health, healthcare, and medicine. IEEE Xplore and ACM Digital Library were searched because these databases index publications in information technology and information management. Web of Science was included because it is a very large database that indexes studies in various disciplines, and also because of its multidisciplinary nature, there exists a consensus among researchers that it is good practice to include this database in systematic reviews. We recognize that Google Scholar is increasingly used as a source for systematic reviews, but that there exists a debate among scientists about its appropriateness [62]. A common argument against Google Scholar is that its algorithm for ranking the relevance of publications is updated frequently, thereby making the search results unreliable for reproduction [62]. Therefore, we have excluded Google Scholar as an information source in this review. Furthermore, because it is a commonality in information technology and computational linguistics that materials are not always published in peer-reviewed journals, but instead it is frequently published only in conference proceedings or conference papers, both journal articles and conference proceedings were included in this systematic review. It was not expected that this would have a significant effect on the reliability of studies, because conference proceedings and conference papers are also subject to a peer-review process.

**Figure 1:**
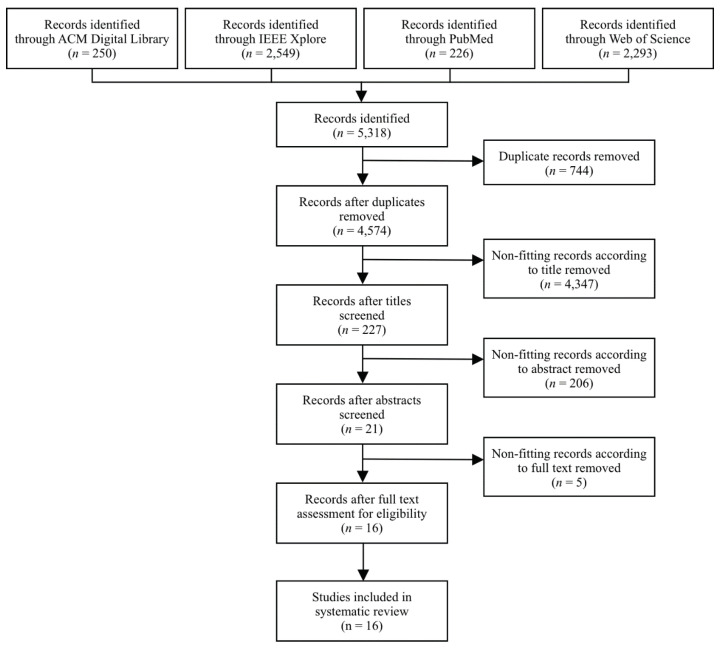
Flow diagram for literature search and study selection.

For each of the included databases, an optimized search strategy was formulated (see Appendix B). The search query was constructed from two blocks. The first block addresses the concept of NLP, and the second block includes search terms related to health surveillance. The systematic literature search was performed on 25 March 2020 for all databases. All publications appearing up to this point were considered in the search. After the databases were searched, the method for de-duplication by Bramer et al. [63] was performed to identify and remove duplicate studies. Studies eligible for this systematic review were selected in three subsequent phases and visualized in Figure 1.

### 3.2. Study Selection

First, the titles were screened for the presence of subjects related to public health monitoring or public health surveillance. The screening was very global to prevent the unnecessary exclusion of studies. Therefore, not only terms such as “adverse drug reactions” were considered relevant, but titles containing more indirect terms such as “medication outcomes” were also included. In addition, if it was ambiguous whether a study was relevant or not, it was still included for further screening in the next phase. Studies that were not relevant were omitted from the library.

Second, the abstracts were screened for information related to NLP, public health monitoring, public health surveillance, and pharmacovigilance. The keywords provided with the manuscript for indexing purposes were also screened for these concepts. This phase was also intended to be broad. For example, abstracts were considered relevant if they contained terms directly related to pharmacovigilance, such as “adverse effects of drug treatment”, but also indirectly related terms such as “drug reviews”. Drug reviews involve an extensive process where experimental drugs are assessed on safety (e.g., toxicity and side effects) and effectiveness using various clinical trials [1]. Drug reviews are mainly performed by pharmaceutical companies which document their tests for review by the European Medicines Agency (EMA) or the U.S. Food and Drug Administration (FDA) [1]. Post market authorization, existing drugs and their side effects are continuously being monitored by medical doctors, laboratories, pharmaceutical organizations, and health authorities [1]. Publications were still included if their relevance was considered ambiguous, for further screening in the next phase. Irrelevant manuscripts were removed.

Third, the full text was downloaded and read. Studies were considered relevant if they investigated the application of NLP to understand text with the purpose of public health monitoring or public health surveillance within the discipline of pharmacovigilance. Eligible studies reported on the application and results of using computational linguistics to identify adverse drug reactions from textual sources, such as forums, patient records, and social media.

### 3.3. Inclusion and Exclusion Criteria

Overall, studies were only eligible for inclusion in this systematic review if they aimed to identify adverse drug reactions using computational linguistics. Both journal articles and conference proceedings were included. In addition, we only included studies if written in the English language, irrespective of the language of the dataset of user-generated content that these studies utilized. There were no limitations regarding the publication date, institutional affiliation, or the journal that these studies were published in.

Publications were excluded if they only reported on a framework instead of the actual application. For example, authors may suggest a process to investigate adverse drug reactions using computational linguistics without actually applying it and evaluating the results. Likewise, studies were excluded if they were published in a language other than English. We only excluded studies if the manuscript was not written in English, irrespective of the language that its dataset was written in. Furthermore, if the same publication was published in different formats, for example as both a conference proceeding and a journal article, only one format of the publication, namely the journal article, was retained.

### 3.4. Reliability and Validity

The included publications were evaluated on quality by assessing their reliability and validity. This assessment was performed using the strategy of Kampmeijer et al. [64]. A publication was evaluated as *reliable* if it reported a thorough and repeatable description of the performed process, methods, data collection, and data analysis [64]. A reliable study provides a well-defined, transparent, and consistent protocol for the collection, processing, and analysis of data. It facilitates researchers to establish its consistency and identify potential flaws in the research design. In addition, a reliable study provides sufficient details such that it can be reproduced. Under the same conditions, if repeated, a reliable study will produce similar findings.

A publication was evaluated as *valid* if the reported findings are logically the result of the described process, methods, data, and analyses that were used to find that result [64]. The validity of a study refers to its accuracy; the study indeed measures what it intended to measure. This evaluation requires that researchers are transparent about their protocol. Assessing the validity of a study involves identifying that the reported results and conclusions in a study are consistent with the study hypotheses and research design. In addition, it involves the verification that the reported findings from one study are comparable to other studies utilizing a comparable research protocol. The identified consistency within one paper, or consistency in reported findings among comparable papers, are indications that a paper was evaluated as valid.

The reliability and validity of studies were assessed qualitatively and discussed among researchers until consensus was achieved. Studies with “low” reliability did not provide a well-defined, transparent, and consistent protocol or this information provided insufficient details. Instead, studies with “high” reliability provided this information and this information was thorough. In all intermediate cases, these studies were marked as “medium” reliability. Similarly, studies with “low” validity had either limited consistency between the hypotheses and research design on the one hand, with the results and conclusions on the other hand, or their findings were not consistent with studies that utilized a comparable research design. Instead, if the consistency was high and their findings were comparable to similar studies, the validity of these studies was marked as “high”. In all intermediate cases, the validity was considered “medium”.

Although the quality assessment was rigorous and based on scientific standards, all identified publications were included in the systematic review.

### 3.5. Data Analysis

Thematic analysis was used to analyze the included publications [65]. The themes were defined by the objectives of the present systematic review. The following themes were extracted from the full text: authors, year of publication, type of drugs, data source, sample size, users, unique users, origin of users, average number of followers, years of data collection, horizon of data collection, software used, techniques and classifiers used, outcome, drugs studied, result, and a description of the result.

For each publication, the extracted themes were processed into an extraction matrix. This matrix was used to synthesize and narratively present the extracted information by theme. The results are summarized and presented using tables.

## 4. Results

The procedure that was followed for the selection of studies is presented in Figure 1. The 5318 initial records, which were identified through an inclusive search strategy, were assessed for the presence of duplicate publications. Consequently, 744 duplicate results were identified and omitted. Therefore, the literature search yielded 4574 unique studies. According to the thorough study selection strategy described in Section 3.2, the first selection phase identified 4347 irrelevant studies to be excluded. In the second phase, the remaining 227 results were screened by reading the abstract; 206 irrelevant studies were omitted. For example, studies were excluded when not mentioning “adverse effects of drug treatment” or other related but rather general terms such as “drug reviews” in the abstract. In the third phase, the full text of the remaining 21 publications was read. Five studies were considered irrelevant because they did not investigate the application of computational linguistics to understand text, with the purpose of public health monitoring or public health surveillance within the discipline of pharmacovigilance.

Overall, this yielded 16 publications that were considered relevant and were included in this systematic review. All studies were published between 2009 and 2019. Most of the studies (69%) were published in the last five years (2015–2019) [31,32,33,34,35,36,37,38,39,40,41]. A summary is provided in Table 1, and it will be further elaborated in the rest of this section.

The reliability and validity of all studies were assessed as medium. While all studies were performed reasonably well, they failed to be entirely transparent about their process, methodology, used software, and the used technologies and classifiers. As is presented in the detailed overview of the characteristics of the included studies in Appendix C, all studies failed to disclose a complete overview of crucial information.

### 4.1. General Characteristics

A general description of the publications included in the analysis is provided in Table 2. To establish differences between them, various characteristics of these publications were compared and the observed differences are presented in Table 3.

Only one study by Adrover et al. [31] discussed the geographical location of users that published the included posts. They report that the users were from Canada, South Africa, the United Kingdom, or the United States [31]. The remaining 15 studies did not disclose the geographical location of users.

Studies disclosing the date of publication of the textual samples (74%) were published between 2004 and 2015 [31,32,36,40,41,66,68,69]. Content published since 2010 was included in more studies compared to content published before 2010. The remaining 26% of studies did not discuss when the posts were published [33,34,35,37,38,39,67,70].

Studies that reported the date of publication of the included content (50%) were used to compute the time horizon of the collected data [31,32,36,40,41,66,68,69]. In 13% of studies, this horizon was one calendar year [36,66]. In 6% of the studies, this horizon was between two and five years [31]. In another 6%, the horizon ranged between 6 and 10 years [32]. In four studies (25%), the time horizon could not be computed because the data were published within the same calendar year [40,41,68,69]. The remaining studies (50%) did not present the date on which the included data were published [33,34,35,37,38,39,67,70]. Therefore, the horizon of data collection could not be computed.

Discounting the studies that did not present the type of drugs that were studied, drugs to treat asthma (5%) [40], cancer (11%) [38,66], cystic fibrosis (5%) [40], depression (5%) [32], HIV (5%) [31], rheumatoid arthritis (5%) [40], and type 2 diabetes (5%) [40] were investigated. In a majority of studies (58%), the type of drugs was not specified [33,34,35,36,37,39,41,67,68,69,70].

The studies also differed with respect to the number of drugs for which ADRs were investigated. Most studies (31%) included posts concerning 20 or more drugs [36,38,39,67,68], followed by 25% that studied between five and nine drugs [32,41,66,69]. Two studies (13%) included between ten and fourteen drugs [40,70], while only one study (6%) addressed less than five drugs [31]. No studies included between 15 and 19 drugs. The remaining 25% of studies did not disclose the number of drugs that were investigated [33,34,35,37].

### 4.2. Input Sources

Publications used data from four sources. A majority of studies (56%) used textual information from social media to extract ADRs [31,33,34,36,37,39,40,41,66,67]. Drug reviews were also a popular source of unstructured data (22%) [35,38,39,40]. Forums (17%) [32,68,70] and electronic health records (6%) [69] were used less often.

There was a wide diversity in the sample size of the posts used, which ranged from 1245 [37] to more than two billion [66] tweets. Three studies (19%) included less than 5000 posts [31,37,41], six publications (38%) used at least 5000 but less than 20,000 posts [32,33,39,67,68,70], and six studies (38%) were performed using more than 20,000 posts [34,35,36,40,66,69]. The sample size was not reported in one study (6%) [38].

Only one study (6%) by Adrover et al. [31] provided contextual information about the background of the publishers of the included posts. In the remaining studies (94%), the background of these users was not disclosed [32,33,34,35,36,37,38,39,40,41,66,67,68,69,70].

The vast majority of studies (88%) did not provide information about the unique number of users that published the analyzed content [32,33,34,35,37,38,39,40,41,66,67,68,69,70]. In only two studies (13%), it was disclosed that less than 5000 users had published the posts [31,36]. Similarly, only one study (6%) discussed that a user had an average of fewer than 5000 followers [31]. The remaining studies (94%) provided no information about this theme [32,33,34,35,36,37,38,39,40,41,66,67,68,69,70].

Most studies (56%) included at least unstructured data from social media [31,33,34,36,37,39,40,41,66,67]. In particular, all of these studies included data from Twitter, while one study additionally used content from YouTube [40]. This study analyzed 42,544 comments from YouTube to identify patient-reported medication outcomes [40]. The majority of these studies reported that ADRs could indeed be extracted from textual content from social media. In addition, drug reviews (22%) [35,38,39,40] and content from forums (17%) [32,68,70] were studied less frequently. For both data sources, ADRs were identified correctly. The least studied source involved electronic health records, for which also positive results were reported [69].

### 4.3. Employed Methods

The studies reported a vast difference in software that was used (see Table 3). In total, 27 software products were discussed. Often, studies also used alternatives for the same type of software. For example, although some studies used Tweepy (3%) [41], Twitter REST API (3%) [37], or Twitter4J (3%) [36] to retrieve data from Twitter using a different programming language, these software products can be aggregated in the type of Twitter API. By frequency, the spelling checker Hunspell was used most often (9%) [33,34,37]. This tool can reduce the dimensionality of NLP tasks by considering various spellings of a single word. For example, misspellings (e.g., “organiezation”) or inconsistent types of English such as “organization” and “organisation”, would then be transformed into the U.S. word “organization”. Notably, three studies (9%) did not present the software that was used [39,67,70].

Likewise, a vast number of 21 different techniques and classifiers were reported. The more generic term “NLP” [36,37,41,67,68] and the more particular task of sentiment analysis [31,33,35,38,41] were mentioned in five studies (12%). In terms of particular techniques, two studies (5%) and one study (2%) reported the use of term-frequency-inverse document frequency (TF-IDF) [32,34] and term document matrix (TDM) [38], respectively. In terms of models, support vector machines (SVM) was used most often in seven studies (17%) to analyze the quantitative features that were extracted from unstructured data [31,33,38,39,41,66,67]. The remaining 5% of the studies did not disclose the techniques and classifiers that were used [40,69].

### 4.4. Study Effectiveness

Although all included studies investigated how NLP can be applied to understand text for pharmacovigilance, and thus all studies investigated reported ADRs, some studies were more explicit than others in discussing their outcome. For example, several studies explicitly disclosed that the outcome was reported ADRs for cancer (6%) [66], reported ADRs for HIV treatment (6%) [31], user sentiment on depression drugs (6%) [32], and user sentiment on cancer drugs (6%) [38]. The remaining studies were rather inconsistent by presenting the outcome as patient satisfaction with drugs (6%) [35], patient-reported medication outcomes (6%) [40], reported ADRs (61%) [33,34,35,36,37,39,41,67,68,69,70], and reported the effectiveness of drugs (6%) [35].

The studies reported consistent evidence that NLP can be successfully used to understand text for the purpose of pharmacovigilance. A vast majority of studies (88%) presented positive results [31,32,33,35,36,37,38,39,40,41,67,68,69,70]. These studies claimed that ADRs could indeed be extracted accurately and reliably from content published by patients. These studies often compared the accuracy of the adverse effects that were extracted from posts against a list of known ADRs, for example, from the medical package insert or from other reliable sources. Only 13% of the studies reported neutral findings [34,66]. No studies reported a negative result.

For example, Nikfarjam et al. [39] addressed the challenges of patients who use informal language and express medical concepts in lay terms, which may obstruct utilizing patients’ digital content for public health monitoring for pharmacovigilance. They developed a system for extracting ADRs from highly informal and unstructured content from both Twitter and a website for drug reviews. They find that it is possible, with reasonably high performance, to extract complex medical concepts from these platforms. In addition, Sampathkumar et al. [68] aimed to identify mentions about ADRs from user-generated content that were published by drug users to healthcare-related forums and to use those mentions for the purpose of pharmacovigilance. They find that it is possible to extract those mentions about ADRs with good performance and that the mentions are consistent with known ADRs. Likewise, Wu et al. [41] developed a pipeline “for collecting, processing, and analyzing tweets to find signals” about ADRs. They were able to identify several well-known ADRs. Furthermore, Yang et al. [70] mined the associations between drugs and the ADRs that patients published to online healthcare communities. These identified associations were then compared to ADR alerts from the U.S. Food and Drug Administration. They find that association mining appears to be promising for the detection of ADRs.

For named diseases, only one study observed neutral effectiveness for oncological drugs [66]. Specifically, Bian et al. [66] developed an approach to identify drug users and extract ADRs concerning cancer from tweets. They used high-performance computing to analyze more than two billion tweets using NLP, and classified tweets using support vector machines. They, however, find that their classification model had limited performance.

There were no significant inconsistencies in the effectiveness of NLP to identify ADRs with respect to the outcome that was under investigation in each study. For a vast majority of the outcomes, ADRs could indeed be established. For the outcome of reported ADRs for cancer, only neutral effectiveness was reported [66]. Although most of the studies that investigated the outcome of reported ADRs observed positive findings, only one study found a neutral result [34]. There were no notable differences in the effectiveness of NLP with respect to the number of drugs that were considered in the publications.

## 5. Discussion

The purpose of this study was to review the existing evidence on the methods and effectiveness of natural language processing to understand user-generated textual content for the purpose of pharmacovigilance.

The first main finding of this systematic review is that the potential of applying NLP for pharmacovigilance looks very promising. Studies included in this systematic review consistently reported positive results on the effectiveness and accuracy of using NLP that is applied to user-generated digital content to identify ADRs. For all diseases investigated, a vast majority of studies reported that the identified ADRs were consistent with the information provided on the medical package insert. For example, Ru et al. [40] analyzed and compared content about patient-reported medication outcomes concerning asthma, cystic fibrosis, rheumatoid arthritis, and type 2 diabetes, published to the social media sites PatientsLikeMe.com, WebMD.com, Twitter, and YouTube. They find that, although advising that more emphasis should be placed on developing more reliable methods for NLP and text mining, social media platforms are indeed suitable and complementary sources for investigating outcomes of medication. In addition, Mishra et al. [38] assessed pharmaceutical oncological drug reviews authored by patients and published to user forums, and they compared the reported drug-related issues with official drug labels. They used support vector machines to classify sentiments about ADRs with good performance. Furthermore, Akay et al. [32] investigated how user-generated content on a depression-related forum can be used for modeling the exchange of information between users about drug-related treatments for depression. They find that it is possible to use NLP on this content to identify the ADRs of these drugs in greater detail, and they confirmed the identified ADRs using medical literature about these drugs. In another study, Androver et al. [31] studied the potential of using user-generated tweets to identify ADRs for HIV-related drugs and to gauge patient sentiments about these drug treatments. They find that the identified ADRs are consistent with well-recognized toxicities.

The second main finding of this systematic review is that some studies also correctly identified ADRs that were previously unknown. In [68], the authors identified increased suicide risk for the drug Singulair, and an increased risk of acute pancreatitis and altered kidney function for the drug Byetta. In both cases, the FDA required the manufacturing companies to conduct an investigation, and update the labels of the products with a warning indicating these risks. This result suggests that NLP may also be used to identify novel ADRs, and it may serve as a suitable tool for pro-active and real-time health surveillance using remote sensing. As such, this automated system may identify trends and periodically report novel insights to policymakers and public health professionals, and it may support and enable these professionals to initiate interventions timely to protect public health and to maintain, and perhaps even increase, the quality of healthcare further [32].

Although this systematic review finds that the application of computational linguistics may be effective for pharmacovigilance, it does not suggest that the traditional system is obsolete and should be replaced by computational linguistics. Instead, it may be worthwhile to apply computational linguistics as a complementary tool to retrieve and process adverse drug reactions that end-users share on the Internet. This information and the insights may be combined with the adverse drug reactions that are reported by medical professionals, with the purpose to achieve a more complete overview of adverse drug reactions. Similarly, computational linguistics may be a suitable tool for the real-time monitoring of adverse drug reactions.

## 6. Limitations

The systematic literature search and study selection were performed by only one researcher. Therefore, it was not possible to establish inter-rater reliability. However, the process of study selection and the included studies were discussed by the authors until consensus was achieved. Nevertheless, it may be possible that this has introduced selection bias, but this could not be verified.

All studies that were included in this systematic review were found to have a medium quality. Quality was operationalized using reliability and validity. The process and assessment of the quality of the included studies were discussed by the authors until consensus was achieved.

It was observed that studies often failed to report information on the themes that were used to extract relevant information (see Appendix C). Consequently, the absence of these data limited the analyses of the studies with respect to their methodology, sample characteristics, and the utilized techniques. In addition, various publications failed to disclose information on the diseases that the identified ADRs were related to. We highly recommend authors disclose this information. This information has great value, among others, to establish the quality of these studies and to enable replicability, but it may also benefit the research community if the methodology and processes are explained in greater detail.

Because it is a commonality in the field of information technology and computational linguistics that findings are not always published in peer-reviewed journals, but instead it is often only published in conference proceedings or conference papers, both types of publications were included in this systematic review. This is important, because it may be possible that the process of peer-review is more rigorous when performed by journals compared to conferences. It was also observed that a significant number of included publications were not journal articles.

A common and unavoidable limitation of studies using user-generated content from social media (including all data sources listed in this review) is the inherent noisiness and bias of these data sources. In the context of the reviewed studies, users are usually unqualified to assess their symptoms, they might exaggerate mild or unrelated symptoms, they might just follow popular trends in criticism, or be biased or even malicious (e.g., seeking to discredit competition). These factors have to be taken into account when judging the effectiveness of the proposed tools.

As we discussed in the methods section, due to the interdisciplinary nature of the reviewed studies and their limitations, it was impossible to apply the complete PRISMA 2020 checklist in this systematic review. Therefore no registration was made in PROSPERO either. We acknowledge this as a limitation of this work.

## 7. Conclusions and Future Outlook

Our findings suggest that the user-generated textual content that drug users share on the Internet may have the potential to augment or enhance the expensive and time-consuming traditional system of pharmacovigilance. NLP may thus be used to automate the monitoring of ADRs using content that users publish to social media and other digital platforms [40]. This novel tool may not only contribute to improving public health and the quality of healthcare, but it could potentially also reduce the costs and processing time that are associated with conducting pharmacovigilance. Therefore, this tool may be a viable solution that addresses two of the most prominent challenges of traditional pharmacovigilance, namely the reduction of the high associated costs [7] and the inclusion of ADRs as experienced by the end-users [6]. It is strongly suggested for policymakers to consider the automated analysis of user-generated textual content for the purpose of pharmacovigilance, and to employ it ethically, responsibly, and with great respect to the privacy and anonymity of these drug users.

We acknowledge that we ought to be limited in describing the architecture of such tools for pharmacovigilance in the present paper. On an abstract level, this tool would subscribe to the Twitter API and filters Tweets based on keywords related to ADRs. Relevant Tweets are then subject to sentiment analysis and processed using NLP techniques. Based on the informational needs of researchers, further processing and analysis can be performed to extract key information on ADRs for medicines of interest and the related sentiment expressed by drug users.

## Figures and Tables

**Table 1:** Summary of characteristics of publications included in the analysis.

Authors	Data Source	Sample Size	Horizon of Data Collection	Software Used	Techniques and Classifiers Used	Outcome	Result	Description of Result
[31]	Social media	Twitter.com: 1642 tweets	3 years	Toolkit for Multivariate Analysis	Artificial Neural Networks (ANN), Boosted Decision Trees with AdaBoost (BDT), Boosted Decision Trees with Bagging (BDTG), Sentiment Analysis, Support Vector Machines (SVM)	Reported ADRs for HIV treatment	Positive	Reported adverse effects are consistent with well-recognized toxicities.
[32]	Forums	DepressionForums.org: 7726 posts	10 years	General Architecture for Text Engineering (GATE), NLTK Toolkit within MATLAB, RapidMiner	Hyperlink-Induced Topic Search (HITS), k-Means Clustering, Network Analysis, Term-Frequency-Inverse Document Frequency (TF-IDF)	User sentiment on depression drugs	Positive	Natural language processing is suitable to extract information on ADRs concerning depression.
[66]	Social media	Twitter.com: 2,102,176,189 tweets	1 year	Apache Lucene	MetaMap, Support Vector Machines (SVM)	Reported ADRs for cancer	Neutral	Classification models had limited performance. Adverse events related to cancer drugs can potentially be extracted from tweets.
[33]	Social media	Twitter.com: 6528 tweets	Unknown	GENIA tagger, Hunspell, Snowball stemmer, Stanford Topic Modelling Toolbox, Twokenizer	Backward/Forward Sequential Feature Selection (BSFS/FSFS) Algorithm, k-Means Clustering, Sentiment Analysis, Support Vector Machines (SVM)	Reported ADRs	Positive	ADRs were identified reasonably well.
[34]	Social media	Twitter.com: 32,670 tweets	Unknown	Hunspell, Twitter tokenizer	Term Frequency-Inverse Document Frequency (TF-IDF)	Reported ADRs	Neutral	ADRs were not identified very well.
[67]	Social media	Twitter.com: 10,822 tweets	Unknown	Unknown	Naive Bayes (NB), Natural Language Processing (NLP), Support Vector Machines (SVM)	Reported ADRs	Positive	ADRs were identified well.
[35]	Drug reviews	Drugs.com, Drugslib.com: 218,614 reviews	Unknown	BeautifulSoup	Logistic Regression, Sentiment Analysis	Patient satisfaction with drugs, Reported ADRs, Reported effectiveness of drugs	Positive	Classification results were very good.
[36]	Social media	Twitter.com: 172,800 tweets	1 year	Twitter4J	Decision Trees, Medical Profile Graph, Natural Language Processing (NLP)	Reported ADRs	Positive	Building a medical profile of users enables the accurate detection of adverse drug events.
[37]	Social media	Twitter.com: 1245 tweets	Unknown	CRF++ Toolkit, GENIA tagger, Hunspell, Twitter REST API, Twokenizer	Natural Language Processing (NLP)	Reported ADRs	Positive	ADRs were identified reasonably well.
[38]	Drug reviews	WebMD.com: Unknown	Unknown	SentiWordNet, WordNet	Sentiment Analysis, Support Vector Machines (SVM), Term document Matrix (TDM)	User sentiment on cancer drugs	Positive	Sentiment on ADRs was identified reasonably well.
[39]	Drug reviews, Social media	DailyStrength.org: 6279 reviews, Twitter.com: 1784 tweets	Unknown	Unknown	ARDMine, Lexicon-based, MetaMap, Support Vector Machines (SVM)	Reported ADRs	Positive	ADRs were identified very well.
[40]	Drug reviews, Social media	PatientsLikeMe.com: 796 reviews, Twitter.com: 39,127 tweets, WebMD.com: 2567 reviews, YouTube.com: 42,544 comments	Not applicable	Deeply Moving	Unknown	Patient-reported medication outcomes	Positive	Social media serves as a new data source to extract patient-reported medication outcomes.
[68]	Forums	Medications.com: 8065 posts, SteadyHealth.com: 11,878	Not applicable	Java Hidden Markov Model library, jsoup	Hidden Markov Model (HMM), Natural Language Processing (NLP)	Reported ADRs	Positive	Reported adverse effects are consistent with well-recognized side-effects.
[69]	Electronic Health Record (EHR)	25,074 discharge summaries	Not applicable	MedLEE	Unknown	Reported ADRs	Positive	Reported adverse effects are consistent with well-recognized toxicities (recall: 75%; precision: 31%).
[41]	Social media	Twitter.com: 3251 tweets	Not applicable	AFINN, Bing Liu sentiment words, Multi-Perspective Question Answering (MPQA), SentiWordNet, TextBlob, Tweepy, WEKA	MetaMap, Naive Bayes (NB), Natural Language Processing (NLP), Sentiment Analysis, Support Vector Machines (SVM)	Reported ADRs	Positive	Several well-known ADRs were identified.
[70]	Forums	MedHelp.org: 6244 discussion threads	Unknown	Unknown	Association Mining	Reported ADRs	Positive	ADRs were identified.

**Table 2:** General description of publications included in the analysis.

Category	Sub-Categories	n (%)	References
Year of publication	2009	1 (6)	[69]
	2010	0 (0)	-
	2011	0 (0)	-
	2012	2 (13)	[66,70]
	2013	0 (0)	-
	2014	2 (13)	[67,68]
	2015	7 (43)	[31,36,37,38,39,40,41]
	2016	2 (13)	[32,33]
	2017	0 (0)	-
	2018	1 (6)	[35]
	2019	1 (6)	[34]
Type of drugs	Asthma	1 (5)	[40]
	Cancer	2 (11)	[38,66]
	Cystic fibrosis	1 (5)	[40]
	Depression	1 (5)	[32]
	HIV	1 (5)	[31]
	Rheumatoid arthritis	1 (5)	[40]
	Type 2 diabetes	1 (5)	[40]
	Unknown	11 (58)	[33,34,35,36,37,39,41,67,68,69,70]
Data source	Drug reviews	4 (22)	[35,38,39,40]
	Electronic Health Records (EHR)	1 (6)	[69]
	Forums	3 (17)	[32,68,70]
	Social media	10 (56)	[31,33,34,36,37,39,40,41,66,67]
Sample size	Less than 5000	3 (19)	[31,37,41]
	5000 to 9999	4 (25)	[32,33,39,70]
	10,000 to 14,999	1 (6)	[67]
	15,000 to 19,999	1 (6)	[68]
	20,000 or more	6 (38)	[34,35,36,40,66,69]
	Unknown	1 (6)	[38]
Users	HIV-infected persons undergoing drug treatment	1 (6)	[31]
	Unknown	15 (94)	[32,33,34,35,36,37,38,39,40,41,66,67,68,69,70]
Unique users	Less than 5000	2 (13)	[31,36]
	5000 to 9999	0 (0)	-
	10,000 to 14,999	0 (0)	-
	15,000 to 19,999	0 (0)	-
	20,000 or more	0 (0)	-
	Unknown	14 (88)	[32,33,34,35,37,38,39,40,41,66,67,68,69,70]
Origin of users	Canada	1 (5)	[31]
	South Africa	1 (5)	[31]
	United Kingdom	1 (5)	[31]
	United States	1 (5)	[31]
	Unknown	15 (79)	[32,33,34,35,36,37,38,39,40,41,66,67,68,69,70]
Average number of followers	Less than 5000	1 (6)	[31]
	5000 to 9999	0 (0)	-
	10,000 to 14,999	0 (0)	-
	15,000 to 19,999	0 (0)	-
	20,000 or more	0 (0)	-
	Unknown	15 (94)	[32,33,34,35,36,37,38,39,40,41,66,67,68,69,70]
Years of data collection	2004	2 (6)	[32,69]
	2005	1 (3)	[32]
	2006	1 (3)	[32]
	2007	1 (3)	[32]
	2008	1 (3)	[32]
	2009	2 (6)	[32,66]
	2010	3 (10)	[31,32,66]
	2011	2 (6)	[31,32]
	2012	3 (10)	[31,32,68]
	2013	2 (6)	[31,32]
	2014	3 (10)	[32,36,40]
	2015	2 (6)	[36,41]
	Unknown	8 (26)	[33,34,35,37,38,39,67,70]
Horizon of data collection	1 year	2 (13)	[36,66]
	2 to 5 years	1 (6)	[31]
	6 to 10 years	1 (6)	[32]
	Not applicable	4 (25)	[40,41,68,69]
	Unknown	8 (50)	[33,34,35,37,38,39,67,70]
Software used	AFINN	1 (3)	[41]
	Apache Lucene	1 (3)	[66]
	BeautifulSoup	1 (3)	[35]
	Bing Liu sentiment words	1 (3)	[41]
	CRF++ toolkit	1 (3)	[37]
	Deeply Moving	1 (3)	[40]
	General Architecture for Text Engineering (GATE)	1 (3)	[32]
	GENIA tagger	2 (6)	[33,37]
	Hunspell	3 (9)	[33,34,37]
	Java Hidden Markov Model library	1 (3)	[68]
	jsoup	1 (3)	[68]
	MedLEE	1 (3)	[69]
	Multi-Perspective Question Answering (MPQA)	1 (3)	[41]
	NLTK toolkit within MATLAB	1 (3)	[32]
	RapidMiner	1 (3)	[32]
	SentiWordNet	2 (6)	[38,41]
	Snowball stemmer	1 (3)	[33]
	Stanford Topic Modelling Toolbox	1 (3)	[33]
	TextBlob	1 (3)	[41]
	Toolkit for Multivariate Analysis	1 (3)	[31]
	Tweepy	1 (3)	[41]
	Twitter REST API	1 (3)	[37]
	Twitter tokenizer	1 (3)	[34]
	Twitter4J	1 (3)	[36]
	Twokenizer	2 (6)	[33,37]
	Unknown	3 (9)	[39,67,70]
	WEKA	1 (3)	[41]
	WordNet	1 (3)	[38]
Techniques and classifiers used	ARDMine	1 (2)	[39]
	Artificial Neural Networks (ANN)	1 (2)	[31]
	Association Mining	1 (2)	[70]
	Backward/forward sequential feature selection (BSFS/FSFS) algorithm	1 (2)	[33]
	Boosted Decision Trees with AdaBoost (BDT)	1 (2)	[31]
	Boosted Decision Trees with Bagging (BDTG)	1 (2)	[31]
	Decision Trees	1 (2)	[36]
	Hidden Markov Model (HMM)	1 (2)	[68]
	Hyperlink-Induced Topic Search (HITS)	1 (2)	[32]
	k-Means Clustering	2 (5)	[32,33]
	Lexicon-based	1 (2)	[39]
	Logistic Regression	1 (2)	[35]
	Medical Profile Graph	1 (2)	[36]
	MetaMap	3 (7)	[39,41,66]
	Naive Bayes (NB)	2 (5)	[41,67]
	Natural Language Processing (NLP)	5 (12)	[36,37,41,67,68]
	Network Analysis	1 (2)	[32]
	Sentiment Analysis	5 (12)	[31,33,35,38,41]
	Support Vector Machines (SVM)	7 (17)	[31,33,38,39,41,66,67]
	Term Document Matrix (TDM)	1 (2)	[38]
	Term-Frequency-Inverse Document Frequency (TF-IDF)	2 (5)	[32,34]
	Unknown	2 (5)	[40,69]
Outcome	Patient satisfaction with drugs	1 (6)	[35]
	Patient-reported medication outcomes	1 (6)	[40]
	Reported ADRs	11 (61)	[33,34,35,36,37,39,41,67,68,69,70]
	Reported ADRs for cancer	1 (6)	[66]
	Reported ADRs for HIV treatment	1 (6)	[31]
	Reported effectiveness of drugs	1 (6)	[35]
	User sentiment on depression drugs	1 (6)	[32]
	User sentiment on cancer drugs	1 (6)	[38]
Drugs studied	Less than 5	1 (6)	[31]
	5 to 9	4 (25)	[32,41,66,69]
	10 to 14	2 (13)	[40,70]
	15 to 19	0 (0)	-
	20 or more	5 (31)	[36,38,39,67,68]
	Unknown	4 (25)	[33,34,35,37]
Result	Positive	14 (88)	[31,32,33,35,36,37,38,39,40,41,67,68,69,70]
	Neutral	2 (13)	[34,66]
	Negative	0 (0)	-
Reliability	Low	0 (0)	-
	Medium	16 (100)	[31,32,33,34,35,36,37,38,39,40,41,66,67,68,69,70]
	High	0 (0)	-
Validity	Low	0 (0)	-
	Medium	16 (100)	[31,32,33,34,35,36,37,38,39,40,41,66,67,68,69,70]
	High	0 (0)	-

**Table 3:** Publications by classification category and result.

Category	Sub-Categories	Positive (n %)	Neutral (n %)	Negative (n %)	References
Type of drugs	Asthma	1 (5)	0 (0)	0 (0)	[40]
	Cancer	1 (5)	1 (5)	0 (0)	[38,66]
	Cystic fibrosis	1 (5)	0 (0)	0 (0)	[40]
	Depression	1 (5)	0 (0)	0 (0)	[32]
	HIV	1 (5)	0 (0)	0 (0)	[31]
	Rheumatoid arthritis	1 (5)	0 (0)	0 (0)	[40]
	Type 2 diabetes	1 (5)	0 (0)	0 (0)	[40]
	Unknown	10 (53)	1 (5)	0 (0)	[33,34,35,36,37,39,41,67,68,69,70]
Data source	Drug reviews	4 (22)	0 (0)	0 (0)	[35,38,39,40]
	Electronic Health Records (EHR)	1 (6)	0 (0)	0 (0)	[69]
	Forums	3 (17)	0 (0)	0 (0)	[32,68,70]
	Social media	8 (44)	2 (11)	0 (0)	[31,33,34,36,37,39,40,41,66,67]
Origin of users	Canada	1 (5)	0 (0)	0 (0)	[31]
	South Africa	1 (5)	0 (0)	0 (0)	[31]
	United Kingdom	1 (5)	0 (0)	0 (0)	[31]
	United States	1 (5)	0 (0)	0 (0)	[31]
	Unknown	13 (68)	2 (11)	0 (0)	[32,33,34,35,36,37,38,39,40,41,66,67,68,69,70]
Horizon of data collection	1 year	1 (6)	1 (6)	0 (0)	[36,66]
	2 to 5 years	1 (6)	0 (0)	0 (0)	[31]
	6 to 10 years	1 (6)	0 (0)	0 (0)	[32]
	Not applicable	4 (25)	0 (0)	0 (0)	[40,41,68,69]
	Unknown	7 (44)	1 (6)	0 (0)	[33,34,35,37,38,39,67,70]
Outcome	Patient satisfaction with drugs	1 (6)	0 (0)	0 (0)	[35]
	Patient-reported medication outcomes	1 (6)	0 (0)	0 (0)	[40]
	Reported ADRs	10 (56)	1 (6)	0 (0)	[33,34,35,36,37,39,41,67,68,69,70]
	Reported ADRs for cancer	0 (0)	1 (6)	0 (0)	[66]
	Reported ADRs for HIV treatment	1 (6)	0 (0)	0 (0)	[31]
	Reported effectiveness of drugs	1 (6)	0 (0)	0 (0)	[35]
	User sentiment on depression drugs	1 (6)	0 (0)	0 (0)	[32]
	User sentiment on cancer drugs	1 (6)	0 (0)	0 (0)	[38]
Drugs studied	Less than 5	1 (6)	0 (0)	0 (0)	[31]
	5 to 9	3 (19)	1 (6)	0 (0)	[32,41,66,69]
	10 to 14	2 (13)	0 (0)	0 (0)	[40,70]
	15 to 19	0 (0)	0 (0)	0 (0)	-
	20 or more	5 (31)	0 (0)	0 (0)	[36,38,39,67,68]
	Unknown	3 (19)	1 (6)	0 (0)	[33,34,35,37]
Reliability	Low	0 (0)	0 (0)	0 (0)	-
	Medium	14 (88)	2 (13)	0 (0)	[31,32,33,34,35,36,37,38,39,40,41,66,67,68,69,70]
	High	0 (0)	0 (0)	0 (0)	-
Validity	Low	0 (0)	0 (0)	0 (0)	-
	Medium	14 (88)	2 (13)	0 (0)	[31,32,33,34,35,36,37,38,39,40,41,66,67,68,69,70]
	High	0 (0)	0 (0)	0 (0)	-

## Data Availability

The resources used in this systematic review are available from the first author.

## Appendix A. PRISMA 2020 Checklist

**Table A1:** PRISMA checklist.

Section/Topic	#	Checklist Item	Reported on Page #
TITLE			
Title	1	Identify the report as a systematic review, meta-analysis, or both.	1
ABSTRACT			
Abstract	2	See the PRISMA 2020 for Abstracts checklist.	1
INTRODUCTION			
Rationale	3	Describe the rationale for the review in the context of what is already known.	1
Objectives	4	Provide an explicit statement of the objective(s) or question(s) the review addresses.	2
METHOD			
Eligibility criteria	5	Specify the inclusion and exclusion criteria for the review and how studies were grouped for the syntheses.	5
Information sources	6	Specify all databases, registers, websites, organisations, reference lists and other sources searched or consulted to identify studies. Specify the date when each source was last searched or consulted.	3
Search strategy	7	Present the full search strategies for all databases, registers and websites, including any filters and limits used.	3, 19
Selection process	8	Specify the methods used to decide whether a study met the inclusion criteria of the review, including how many reviewers screened each record and each report retrieved, whether they worked independently, and if applicable, details of automation tools used in the process.	4
Data collection process	9	Specify the methods used to collect data from reports, including how many reviewers collected data from each report, whether they worked independently, any processes for obtaining or confirming data from study investigators, and if applicable, details of automation tools used in the process.	4
Data items	10a	List and define all outcomes for which data were sought. Specify whether all results that were compatible with each outcome domain in each study were sought (e.g. for all measures, time points, analyses), and if not, the methods used to decide which results to collect.	4
	10b	List and define all other variables for which data were sought (e.g. participant and intervention characteristics, funding sources). Describe any assumptions made about any missing or unclear information.	4, 5
Study risk of bias assessment	11	Specify the methods used to assess risk of bias in the included studies, including details of the tool(s) used, how many reviewers assessed each study and whether they worked independently, and if applicable, details of automation tools used in the process.	5
Effect measures	12	Specify for each outcome the effect measure(s) (e.g. risk ratio, mean difference) used in the synthesis or presentation of results.	n. a.
Synthesis methods	13a	Describe the processes used to decide which studies were eligible for each synthesis (e.g. tabulating the study intervention characteristics and comparing against the planned groups for each synthesis (item #5)).	5
	13b	Describe any methods required to prepare the data for presentation or synthesis, such as handling of missing summary statistics, or data conversions.	6
	13c	Describe any methods used to tabulate or visually display results of individual studies and syntheses.	6
	13d	Describe any methods used to synthesize results and provide a rationale for the choice(s). If meta-analysis was performed, describe the model(s), method(s) to identify the presence and extent of statistical heterogeneity, and software package(s) used.	6
	13e	Describe any methods used to explore possible causes of heterogeneity among study results (e.g. subgroup analysis, meta-regression).	n. a.
	13f	Describe any sensitivity analyses conducted to assess robustness of the synthesized results.	n. a.
Reporting bias assessment	14	Describe any methods used to assess risk of bias due to missing results in a synthesis (arising from reporting biases).	n. a.
Certainty assessment	15	Describe any methods used to assess certainty (or confidence) in the body of evidence for an outcome.	5
RESULTS			
Study selection	16a	Describe the results of the search and selection process, from the number of records identified in the search to the number of studies included in the review, ideally using a flow diagram.	4
	16b	Cite studies that might appear to meet the inclusion criteria, but which were excluded, and explain why they were excluded.	n. a.
Study characteristics	17	Cite each included study and present its characteristics.	7
Risk of bias within studies	18	Present assessments of risk of bias for each included study.	20
Results of individual studies	19	For all outcomes, present, for each study: (a) summary statistics for each group (where appropriate) and (b) an effect estimate and its precision (e.g. confidence/credible interval), ideally using structured tables or plots.	9
Results of syntheses	20a	For each synthesis, briefly summarise the characteristics and risk of bias among contributing studies.	n. a.
	20b	Present results of all statistical syntheses conducted. If meta-analysis was done, present for each the summary estimate and its precision (e.g. confidence/credible interval) and measures of statistical heterogeneity. If comparing groups, describe the direction of the effect.	n. a.
	20c	Present results of all investigations of possible causes of heterogeneity among study results.	n. a.
	20d	Present results of all sensitivity analyses conducted to assess the robustness of the synthesized results.	n. a.
Reporting biases	21	Present assessments of risk of bias due to missing results (arising from reporting biases) for each synthesis assessed.	15
Certainty of evidence	22	Present assessments of certainty (or confidence) in the body of evidence for each outcome assessed.	n. a.
DISCUSSION			
Discussion	23a	Provide a general interpretation of the results in the context of other evidence.	14
	23b	Discuss any limitations of the evidence included in the review.	15
	23c	Discuss any limitations of the review processes used.	15
	23d	Discuss implications of the results for practice, policy, and future research.	16
OTHER INFORMATION			
Registration and protocol	24a	Provide registration information for the review, including register name and registration number, or state that the review was not registered.	15
	24b	Indicate where the review protocol can be accessed, or state that a protocol was not prepared.	15
	24c	Describe and explain any amendments to information provided at registration or in the protocol.	15
Support	25	Describe sources of financial or non-financial support for the review, and the role of the funders or sponsors in the review.	16
Competing interests	26	Declare any competing interests of review authors.	16
Availability of data, code and other materials	27	Report which of the following are publicly available and where they can be found: template data collection forms; data extracted from included studies; data used for all analyses; analytic code; any other materials used in the review.	n. a.

## Appendix B. Search Strategies

The systematic literature search was performed on 25 March 2020 for all databases. All publications appearing up to this point were considered in the search.

### Appendix B.1. Search Strategy for PubMed

Block 1: Computational Linguistics

artificial intelligence[Title/Abstract] OR Artificial Intelligence[MeSH Terms] OR machine learning[Title/Abstract] OR Machine Learning[MeSH Terms] OR text mining[Title/ Abstract] OR computational linguistics[Title/Abstract] OR natural language processing[Title/ Abstract] OR Natural Language Processing[MeSH Terms] OR nlp[Title/Abstract] OR sentiment analysis[Title/Abstract] OR word embedding*[Title/Abstract] OR natural language toolkit[Title/Abstract] OR nltk[Title/Abstract]

Block 2: Health Surveillance

public health surveillance[Title/Abstract] OR Public Health Surveillance[MeSH Terms] OR health surveillance[Title/Abstract] OR public health monitoring[Title/Abstract] OR health monitoring[Title/Abstract]

Filters:

Article types: Journal Article

Languages: English

### Appendix B.2. Search Strategy for Web of Science

Block 1: Computational Linguistics

TS=(artificial intelligence) OR TS=(machine learning) OR TS=(text mining) OR TS=(computational linguistics) OR TS=(natural language processing) OR TS=(nlp) OR TS=(sentiment analysis) OR TS=(word embedding*) OR TS=(natural language toolkit) OR TS=(nltk)

Block 2: Health Surveillance

TS=(public health surveillance) OR TS=(health surveillance) OR TS=(public health monitoring) OR TS=(health monitoring)

Filters:

Document types: Article, Proceedings Paper

Languages: English

### Appendix B.3. Search Strategy for IEEE Xplore

Block 1: Computational Linguistics

“All Metadata”:artificial intelligence OR “All Metadata”:machine learning OR “All Metadata”:text mining OR “All Metadata”:computational linguistics OR “All Metadata”: natural language processing OR “All Metadata”:nlp OR “All Metadata”:sentiment analysis OR “All Metadata”:word embedding* OR “All Metadata”:natural language toolkit OR “All Metadata”:nltk

Block 2: Health Surveillance

“All Metadata”:public health surveillance OR “All Metadata”:health surveillance OR “All Metadata”:public health monitoring OR “All Metadata”:health monitoring

Filters:

Document types: Conferences, Journals

### Appendix B.4. Search Strategy for ACM Digital Library

Block 1: Computational Linguistics

artificial intelligence OR machine learning OR text mining OR computational linguistics OR natural language processing OR nlp OR sentiment analysis OR word embedding* OR natural language toolkit OR nltk

Block 2: Health Surveillance

public health surveillance OR health surveillance OR public health monitoring OR health monitoring

Filters:

Searched within: Abstract, Author Keyword, Title All publications: Proceedings, Research Article

## Appendix C. Characteristics of Publications

**Table A2:** Characteristics of publications included in the analysis.

Authors	Type of Drugs	Drugs Studied	Data Source	Sample Size	Users	Unique Users	Origin of Users	Average Number of Followers	Years of Data Collection	Horizon of Data Collection	Software Used	Techniques and Classifiers Used	Outcome	Result	Description of Result	Reliability	Validity
[31]	HIV	Atripla, Sustiva, Truvada	Social media	Twitter.com: 1642 tweets	HIV-infected persons undergoing drug treatment	512 (male: 247; female: 83; unknown: 182)	Canada, South Africa, United Kingdom, United States (New York City, San Fransisco)	2300	2010, 2011, 2012, 2013	3 years	Toolkit for Multivariate Analysis	Artificial Neural Networks (ANN), Boosted Decision Trees with AdaBoost (BDT), Boosted Decision Trees with Bagging (BDTG), Sentiment Analysis, Support Vector Machines (SVM)	Reported ADRs for HIV treatment	Positive	Reported adverse effects are consistent with well-recognized toxicities.	Medium	Medium
[32]	Depression	Citalopram, Chlorpromazine, Cyclobenzaprine, Dulexetine, Promethazine	Forums	Depression Forums.org: 7726 posts	Unknown	Unknown	Unknown	Unknown	2004–2014	10 years	General Architecture for Text Engineering (GATE), NLTK Toolkit within MATLAB, RapidMiner	Hyperlink-Induced Topic Search (HITS), k-Means Clustering, Network Analysis, Term-Frequency-Inverse Document Frequency (TF-IDF)	User sentiment on depression drugs	Positive	Natural language processing is suitable to extract information on ADRs concerning depression.	Medium	Medium
[66]	Cancer	Avastin, Melphalan, Rupatadin, Tamoxifen, Taxotere	Social media	Twitter.com: 2,102,176,189 tweets	Unknown	Unknown	Unknown	Unknown	2009, 2010	1 year	Apache Lucene	MetaMap, Support Vector Machines (SVM)	Reported ADRs for cancer	Neutral	Classification models had limited performance. Adverse events related to cancer drugs can potentially be extracted from tweets.	Medium	Medium
[33]	Unknown	Unknown	Social media	Twitter.com: 6528 tweets	Unknown	Unknown	Unknown	Unknown	Unknown	Unknown	GENIA tagger, Hunspell, Snowball stemmer, Stanford Topic Modelling Toolbox, Twokenizer	Backward/forward sequential feature selection (BSFS/FSFS) algorithm, k-Means Clustering, Sentiment Analysis, Support Vector Machines (SVM)	Reported ADRs	Positive	ADRs were identified reasonably well.	Medium	Medium
[34]	Unknown	Unknown	Social media	Twitter.com: 32,670 tweets	Unknown	Unknown	Unknown	Unknown	Unknown	Unknown	Hunspell, Twitter tokenizer	- Term Frequency-Inverse Document Frequency (TF-IDF)	Reported ADRs	Neutral	ADRs were not very well identified.	Medium	Medium
[67]	Unknown	65 drugs	Social media	Twitter.com: 10,822 tweets	Unknown	Unknown	Unknown	Unknown	Unknown	Unknown	Unknown	Naive Bayes (NB), Natural Language Processing (NLP), Support Vector Machines (SVM)	Reported ADRs	Positive	ADRs were identified good.	Medium	Medium
[35]	Unknown	Unknown	Drug reviews	Drugs.com, Drugslib.com: 218,614 reviews	Unknown	Unknown	Unknown	Unknown	Unknown	Unknown	BeautifulSoup	Logistic Regression, Sentiment Analysis	Patient satisfaction with drugs, Reported ADRs, Reported effectiveness of drugs	Positive	Classification results were very good.	Medium	Medium
[36]	Unknown	103 drugs	Social media	Twitter.com: 172,800 tweets	Unknown	864	Unknown	Unknown	2014, 2015	1 year	Twitter4J	Decision Trees, Medical Profile Graph, Natural Language Processing (NLP)	Reported ADRs	Positive	Building a medical profile of users enables the accurate detection of adverse drug events.	Medium	Medium
[37]	Unknown	Unknown	Social media	Twitter.com 1245 tweets	Unknown	Unknown	Unknown	Unknown	Unknown	Unknown	CRF++ Toolkit, GENIA tagger, Hunspell, Twitter REST API, Twokenizer	Natural Language Processing (NLP)	Reported ADRs	Positive	ADRs were identified reasonably well.	Medium	Medium
[38]	Cancer	146 drugs	Drug reviews	WebMD.com: Unknown	Unknown	Unknown	Unknown	Unknown	Unknown	Unknown	SentiWordNet, WordNet	Sentiment Analysis, Support Vector Machines (SVM), Term document Matrix (TDM)	User sentiment on cancer drugs	Positive	Sentiment on ADRs was identified reasonably well.	Medium	Medium
[39]	Unknown	81 drugs	Drug reviews, Social media	DailyStrength.org: 6279 reviews, Twitter.com: 1784 tweets	Unknown	Unknown	Unknown	Unknown	Unknown	Unknown	Unknown	ARDMine, Lexicon-based, MetaMap, Support Vector Machines (SVM)	Reported ADRs	Positive	ADRs were identified very well.	Medium	Medium
[40]	Asthma, Cystic fibrosis, Rheumatoid arthritis, Type 2 diabetes	Albuterol, Azithromycin, Bromocriptine, Insulin, Ipratropium, Ivacaftor, Meloxicam, Metformin, Prednisone, Sulfasalazine	Drug reviews, Social media	PatientsLikeMe.com: 796 reviews, Twitter.com: 39,127 tweets, WebMD.com: 2567 reviews, YouTube.com: 42,544 comments	Unknown	Unknown	Unknown	Unknown	2014	Not applicable	Deeply Moving	Unknown	Patient-reported medication outcomes	Positive	Social media serves as a new data source to extract patient-reported medication outcomes.	Medium	Medium
[68]	Unknown	Medications.com: 168 drugs, SteadyHealth.com: 316 drugs	Forums	Medications.com: 8065 posts, SteadyHealth.com: 11,878	Unknown	Unknown	Unknown	Unknown	2012	Not applicable	Java Hidden Markov Model library, jsoup	Hidden Markov Model (HMM), Natural Language Processing (NLP)	Reported ADRs	Positive	Reported adverse effects are consistent with well-recognized side-effects.	Medium	Medium
[69]	Unknown	ACE inhibitors, Bupropion, Ibuprofen, Morphine, Paroxetine, Rosiglitazone, Warfarin	Electronic Health Record (EHR)	25,074 discharge summaries	Unknown	Unknown	Unknown	Unknown	2004	Not applicable	MedLEE	Unknown	Reported ADRs	Positive	Reported adverse effects are consistent with well-recognized toxicities (recall: 75%; precision: 31%).	Medium	Medium
[41]	Unknown	Baclofen, Duloxetine, Gabapentin, Glatiramer, Pregabalin	Social media	Twitter.com: 3251 tweets	Unknown	Unknown	Unknown	Unknown	2015	Not applicable	AFINN, Bing Liu sentiment words, Multi-Perspective Question Answering (MPQA), SentiWordNet, TextBlob, Tweepy, WEKA	MetaMap, Naive Bayes (NB), Natural Language Processing (NLP), Sentiment Analysis, Support Vector Machines (SVM)	Reported ADRs	Positive	Several well-known ADRs were identified.	Medium	Medium
[70]	Unknown	Adenosine, Biaxin, Cialis, Elidel, Lansoprazole, Lantus, Luvox, Prozac, Tacrolimus, Vyvanse	Forums	MedHelp.com: 6244 discussion threads	Unknown	Unknown	Unknown	Unknown	Unknown	Unknown	Unknown	Association Mining	Reported ADRs	Positive	ADRs were identified.	Medium	Medium
